# Early versus delayed EUS-guided drainage for postoperative pancreatic fluid collections: a systematic review and meta-analysis

**DOI:** 10.1007/s00464-023-10568-y

**Published:** 2023-11-28

**Authors:** Tsuyoshi Mukai, Yousuke Nakai, Tsuyoshi Hamada, Saburo Matsubara, Takashi Sasaki, Hirotoshi Ishiwatari, Susumu Hijioka, Hideyuki Shiomi, Mamoru Takenaka, Takuji Iwashita, Atsuhiro Masuda, Tomotaka Saito, Hiroyuki Isayama, Ichiro Yasuda, for the WONDERFUL Study Group in Japan, Tatsuya Sato, Keito Nakagawa, Kentaro Suda, Ryota Nakano, Shogo Ota, Kotaro Takeshita, Shunsuke Omoto, Senju Akihiko, Ryuichi Tezuka, Shinya Uemura, Masahiro Tsujimae, Arata Sakai, Mitsuru Okuno, Yuhei Iwasa, Keisuke Iwata, Kensaku Yoshida, Akinori Maruta, Toshio Fujisawa, Sho Takahashi, Nobuhiko Hayashi

**Affiliations:** 1 Department of Gastroenterological Endoscopy, Kanazawa Medical University, 1-1 Daigaku, Uchinada, Kahoku, Ishikawa 920-0293 Japan; 2 Department of Gastroenterology, Graduate School of Medicine, The University of Tokyo, Tokyo, Japan; 3 Department of Endoscopy and Endoscopic Surgery, The University of Tokyo Hospital, 7-3-1 Hongo, Bunkyo-Ku, Tokyo, 113-8655 Japan; 4 Department of Hepato-Biliary-Pancreatic Medicine, The Cancer Institute Hospital of Japanese Foundation for Cancer Research, Tokyo, Japan; 5 Department of Gastroenterology and Hepatology, Saitama Medical Center, Saitama Medical University, Saitama, Japan; 6 Division of Endoscopy, Shizuoka Cancer Center, Shizuoka, Japan; 7 Department of Hepatobiliary and Pancreatic Oncology, National Cancer Center Japan, Tokyo, Japan; 8 Division of Gastroenterology and Hepatobiliary and Pancreatic Diseases, Department of Internal Medicine, Hyogo Medical University, Hyogo, Japan; 9 Department of Gastroenterology and Hepatology, Faculty of Medicine, Kindai University, Osaka, Japan; 10 First Department of Internal Medicine, Gifu University Hospital, Gifu, Japan; 11 Division of Gastroenterology, Department of Internal Medicine, Kobe University Graduate School of Medicine, Hyogo, Japan; 12 Department of Gastroenterology, Graduate School of Medicine, Juntendo University, Tokyo, Japan; 13 Third Department of Internal Medicine, University of Toyama, Toyama, Japan

**Keywords:** Drainage, Endoscopic ultrasound, Fluid collection, Postoperative pancreatic fistula

## Abstract

**Background:**

Postoperative pancreatic fluid collections (POPFCs) are common adverse events (AEs) after pancreatic surgery and may need interventions. Endoscopic ultrasound (EUS)-guided drainage for POPFCs is increasingly reported, but its appropriate timing has not been fully elucidated. The aim of this meta-analysis was to evaluate treatment outcomes of POPFCs according to the timing of EUS-guided drainage.

**Methods:**

Using PubMed, Embase, Web of Science, and the Cochrane database, we identified clinical studies published until December 2022 with data comparing outcomes of early and delayed EUS-guided drainage for POPFCs. We pooled data on AEs, mortality, and technical and clinical success rates, using the random-effects model.

**Results:**

From 1415 papers identified in the initial literature search, we identified 6 retrospective studies, including 128 and 107 patients undergoing early and delayed EUS-guided drainage for POPFCs. The threshold of early and delayed drainage ranged from 14 to 30 days. Distal pancreatectomy was the major cause of POPFCs, ranging from 44 to 100%. The pooled odds ratio (OR) for AEs was 0.81 (95% confidence interval [CI] 0.40–1.64, *P* = 0.55) comparing early to delayed drainage. There was no procedure-related mortality. Technical success was achieved in all cases and a pooled OR of clinical success was 0.60 (95% CI 0.20–1.83, *P* = 0.37).

**Conclusion:**

POPFCs can be managed by early EUS-guided drainage without an increase in AEs.

**Supplementary Information:**

The online version contains supplementary material available at 10.1007/s00464-023-10568-y.

Postoperative pancreatic fistula (POPF) is a common adverse event (AE) after pancreatic surgery with the reported incidences of up to 25% in pancreaticoduodenectomy [1] and 43% in distal pancreatectomy [2]. Although some POPFs resolve with conservative treatment, interventions such as drainage for infected fluid collection and angiography with embolization for bleeding are necessary in cases with grade B (persistent drainage > 3 weeks and change in the management, including additional non-surgical interventions) and grade C (reoperation or organ failure) POPFs [3]. Historically, symptomatic postoperative pancreatic fluid collections (POPFCs) were managed by surgery or percutaneous drainage, but endoscopic ultrasonography (EUS)-guided approach is increasingly utilized due to its safety and effectiveness reported in pancreatic fluid collections (PFCs) after acute pancreatitis [4, 5]. A recent meta‑analysis has demonstrated a significantly higher clinical success rate and a lower recurrence rate with EUS-guided drainage compared to percutaneous approach in the management of POPFCs [6].

In PFCs due to acute pancreatitis, the timing of interventions is the matter of debate [7–9]. Delayed interventions after four weeks of acute pancreatitis onset have been recommended [10], but the role of early and proactive non-surgical interventions was increasingly investigated [11, 12]. While early interventions for POPFCs may lead to early recovery, it may increase AEs such as bleeding or infection due to the direct intervention within the cavity and potential contamination with gut microbiota. However, data on the timing of interventions are limited in POPFCs, compared to PFCs after acute pancreatitis, and the optimal timing of EUS-guided drainage for POPFCs has not been elucidated. Thus, we conducted a systematic review and meta-analysis to evaluate clinical outcomes of early EUS-guided drainage for POPFCs.

## Methods

### Study overview

This systematic review and meta-analysis aimed to evaluate treatment outcomes of early vs. delayed EUS-guided drainage of POPFCs and was conducted in accordance with the PRISMA (the Preferred Reporting Items for Systematic reviews and Meta-Analyses) guideline [13]. The protocol was registered in the database of UMIN (University Hospital Medical Information Network; registration number, UMIN000049891). This study was conducted by the WONDERFUL (WON anD pERipancreatic FlUid coLlection) study group, which consisted of expert endoscopists, gastroenterologists, interventional radiologists, and epidemiologists at high-volume centers in Japan (UMIN-CTR Registration Number UMIN000044130) [14, 15].

### Literature search

Based on a systematic electronic search using PubMed, Embase, Web of Science, and the Cochrane Central Register of Controlled Trials (CENTRAL) database, we identified clinical studies published from January 1990 through December 2022, in which treatment outcomes were reported in relation to the timing of EUS-guided drainage for POPFCs. The timing of drainage was classified as early or delayed with the cut-off point of 14–30 days of surgery. Two authors (Y.N. and T.M.) independently participated in the literature search, study selection, assessment of study quality, and data extraction. Disagreements were resolved through discussions with another author (T.H.). The search terms included “endoscopy,” “endoscopic,” “EUS,” “pancreatic fistula,” “fluid collection,” “postoperative,” “postsurgical,” “pancreatectomy,” “pancreatic surgery,” “drainage,” “stent,” “treatment,” and “management,” with their word variations (the search strategy in each database was detailed in Supplementary Table 1). The search was limited to fully published articles in English and human studies. The search was not limited in terms of patients’ age and length of patient follow-up. The bibliographies of the identified articles were further screened for additional eligible articles.

The quality of reporting data stratified by the timing of EUS-guided drainage for PFCs was assessed using the Newcastle–Ottawa Scale [16], which ranges from 0 (poor quality) to 9 (good quality) summing up the scores for the following three categories: selection of exposed and non-exposed cohorts (4 points), comparability of cohorts (2 points), and assessment of outcome (3 points). The scores of the included studies are presented in Supplementary Table 2.

### Selection criteria

Based on PICO (population, intervention, comparison, outcome) strategy, studies were selected according to the following inclusion and exclusion criteria.

Inclusion criteria are as follows:

- Population: Patients with POPFC.
- Intervention: EUS-guided drainage for POPFCs with early drainage.
- Comparison: EUS-guided drainage for POFPCs with delayed drainage.
- Outcomes: AEs, procedure-related mortality, technical success, clinical success the number of interventions, length of hospital stay, and recurrence.

Exclusion criteria are studies involving < 10 patients per study and < 2 patients per group, studies examining PFCs due to acute pancreatitis or trauma, and those reporting treatment outcomes only for surgical or percutaneous management of POPFCs.

### Data collection

Using a pre-defined standardized data extraction form, the following data were collected from each study: study design, patient demographics, treatment protocols, and treatment outcomes. The primary endpoint was AEs, and secondary endpoints were procedure-related mortality, technical success, clinical success, the number of interventions, length of hospital stay, and recurrence. AEs included bleeding, infection, stent migration, thrombosis, and abdominal symptoms (persistent pain, nausea, and vomiting).

### Statistical analysis

Using the data reported in the pooled studies, we calculated pooled odds ratios (ORs) and 95% confidence intervals (CIs) for outcome variables comparing early to delayed EUS-guided drainage. Given heterogeneity in study populations and procedures between the studies, we used the DerSimonian–Laird random-effects model [17]. Statistical heterogeneity in outcome variables between the studies was assessed by the Q and *I*^*2*^ statistics [18]. For the Q statistic, we used a *P* value of 0.10 for statistical significance in view of the low power of tests for heterogeneity [19]. The *I*^*2*^ statistics of around 25%, 50%, and 75% were considered as low-, moderate-, and high-level heterogeneity, respectively [20]. We assessed potential publication bias by means of the visual inspection of the funnel plot with the Begg’s rank correlation test [21] and the Egger’s linear regression test [22].

A two-sided *P* value < 0.05 was considered statistically significant. Given multiple comparisons, the results of the secondary analyses were interpreted cautiously. All analyses were performed using R software version 4.1.3 and the metapackage (R Development Core Team, http://www.r-project.org).

## Results

### Study selection

**Fig. 1:**
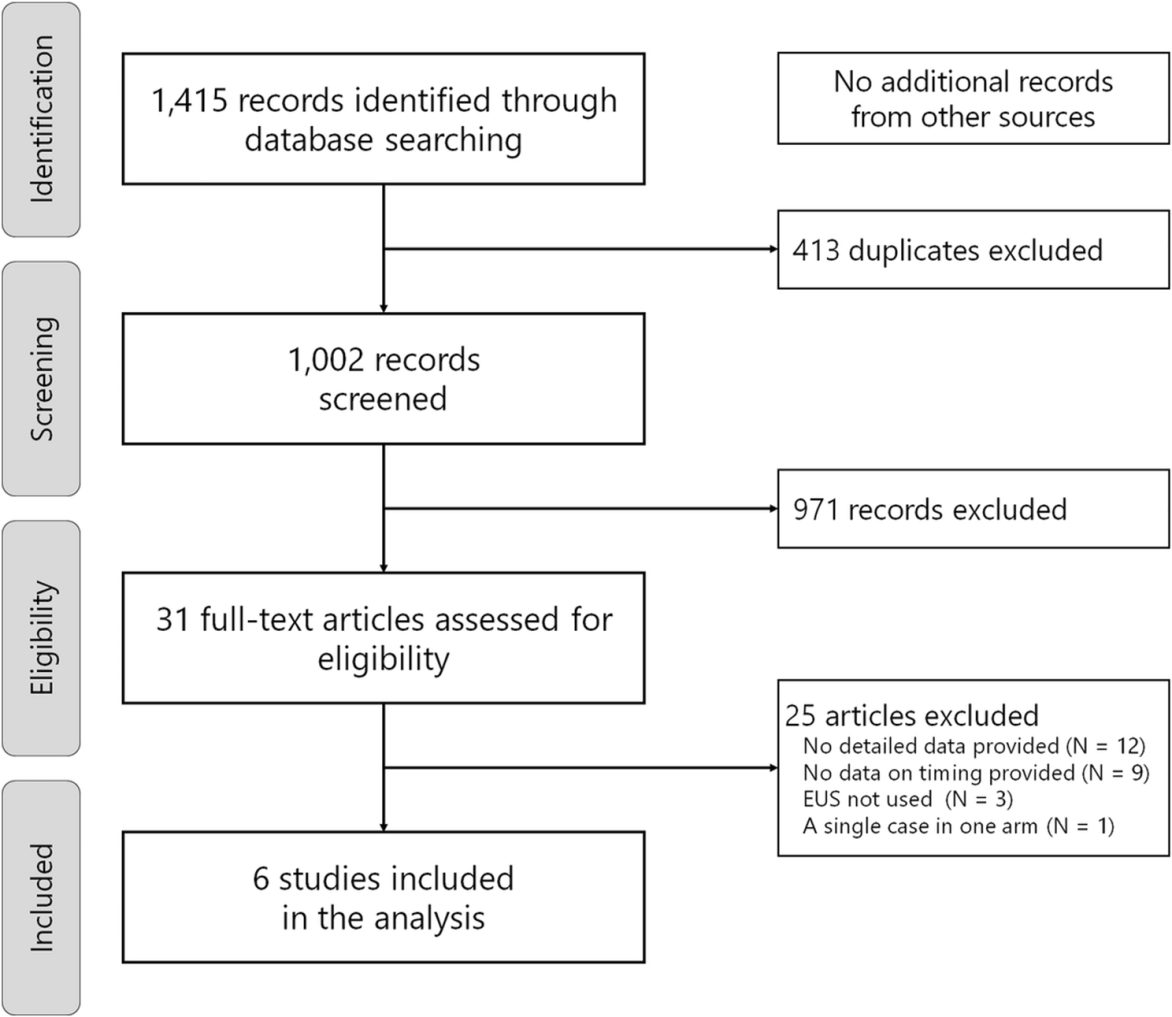
Flowchart of study selection for a meta-analysis of early and delayed EUS-guided interventions for postoperative pancreatic fluid collections

Through the systematic literature search (Fig. 1), we identified six eligible studies [23–28], in which clinical outcomes could be compared by the timing of EUS-guided drainage. The total number of cases included in the analysis was 235 (128 in the early drainage group and 107 in the delayed drainage group), and all studies were conducted based on the retrospective design.

### Study characteristics

The characteristics and clinical outcomes of the included studies are summarized in Tables 1 and 2. Distal pancreatectomy was the major cause of POPFCs, ranging from 44 to 100%, and the mean size of POPFCs was 70 to 95 mm. Abdominal pain and fever were common reasons for EUS-guided drainage. Lumen-apposing metal stents were used in a total of 44 cases in 3 studies [25–27]. While Storm et al. [26] reported the rates of infection at EUS-guided drainage were 41% and 61% in early and delayed drainage groups (*P* = 0.11), respectively, Fujimori et al. [27] reported the corresponding rates of 100% and 31% (*P* < 0.01). Two studies reported the rate of encapsulation of POPFCs, and encapsulation was more likely to be observed in delayed drainage group: 57% vs. 94% (*P* = 0.02) [27] and 72% vs. 95% (*P* = 0.07) [28] in early and delayed drainage groups, respectively.

**Table 1:** Summary of studies included in the meta-analysis

Study	*N*	Threshold for timing of drainage	Early/delayed drainage	Age, years	Sex, male	Surgery, DP	Symptoms	Size, mm	Stents,			Transgastric drainage	Encapsulation	Follow-up period
									PS	SEMS	LAMS			
Varadarajulu [23]	10	30 days^a^	4(40)/6(60)	58 (20–76)	6(60)	10(100)	Abdominal pain 10(100), fever 6(60), leukocytosis 8(80)	95 (45–140)	10(100)	0	0	9(90)	NA	155 (96–280) days
Tilara [24]	31	30 days	17(55)/14(45)	61 (20–83)	13(42)	15(48)	Abdominal pain 26(84), fever 13(42), leukocytosis 3(10), increased size 1(3)	85^b^ (30–150)	31(100)	0	0	30(97)	NA	NA
Caillol [25]	41	25 days	22(54)/19(46)	61^b^	19(46)	26(63)	Abdominal pain 39(95), fever 23(56), increased size 2(5)	76^b^	39(95)^c^	0	2(5)	39(95)	NA	44.75 (29.24–65.74) months
Storm [26]	75	30 days	42(56)/33(44)	59.5^b^	41(53)	63(82)	NA	78^b^	35(47)	2(3)	38(51)	69(92)	NA	262 (39–1485) days
Fujimori [27]	30	15 days	14(47)/16(53)	64.5 (10–87)	18(60)	24(80)	Abdominal pain 7(23), fever 19(63), asymptomatic 2(7), others 2(7)	69.5 (38–145)	23(77)^d^	0	4(13)	29(97)	23(70)	14 (0.6–117) months
Oh [28]	48	14 days	29(60)/19(40)	59.4 (52–69)^e^	30(63)	21(44)	Abdominal pain 27(56), fever 18(38), leukocytosis 2(4), increased size 1(2)	NA	7(15)^c^	41(85)	0	48(100)	39(81)	13.1 (8.1–31.2)^e^ months

Numbers are shown in median (range) or *n* (%), unless otherwise noted

*DP* distal pancreatectomy, *LAMS* lumen-apposing metal stent, *NA* not available, *PS* plastic stent, *SEMS* tubular self-expandable metal stent

^a^Threshold was not originally defined but was set at 30 days due to the balance of case numbers

^b^Mean

^c^Including one nasocystic drain alone

^d^Three patients underwent aspiration alone

^e^Interquartile range

**Table 2:** Clinical outcomes of early and delayed EUS-guided drainage for postoperative pancreatic fluid collection

Study	Group	*N*	Technical success	Clinical success	Adverse event	Bleeding	Infection	Stent migration	Number of interventions	Hospital stay, days	Recurrence
Varadarajulu [23]	Early	4	4(100)	4(100)	0	0	0	0	NA	2.5^a^	0
	Delayed	6	6(100)	5(83)	1(17)	0	0	1(17)	NA	6.2^a^	0
Tilara [24]	Early	17	17(100)	17(100)	1(6)	1(6)	0	0	NA	NA	NA
	Delayed	14	14(100)	14(100)	1(7)	1(7)	0	0	NA	NA	NA
Caillol [25]	Early	22	22(100)	19(86)	10(45)	6(27)	3(14)	0	NA	NA	0
	Delayed	19	19(100)	19(100)	9(47)	3(16)	4(21)	2(11)	NA	NA	0
Storm [26]	Early	42	42(100)	39(93)	9(21)	2(5)	0	0	2^a^	2(1–17)	2(5)
	Delayed	33	33(100)	31(94)	10(30)	2(6)	1(3)	1(3)	2.4^a^	2(1–15)	2(6)
Fujimori [27]	Early	14	14(100)	13(93)	1(7)	1(7)	0	0	1.3^a^	23.5^a^	0
	Delayed	16	16(100)	16(100)	1(6)	1(6)	0	0	1.3^a^	21.4^a^	0
Oh [28]	Early	29	29(100)	28(97)	2(7)	2(7)	0	0	NA	NA	0
	Delayed	19	19(100)	18(95)	0	0	0	0	NA	NA	0

Numbers are shown in median (range) or *n* (%), unless otherwise noted

*NA* not available

^a^Mean

### Adverse events and mortality

**Fig. 2:**
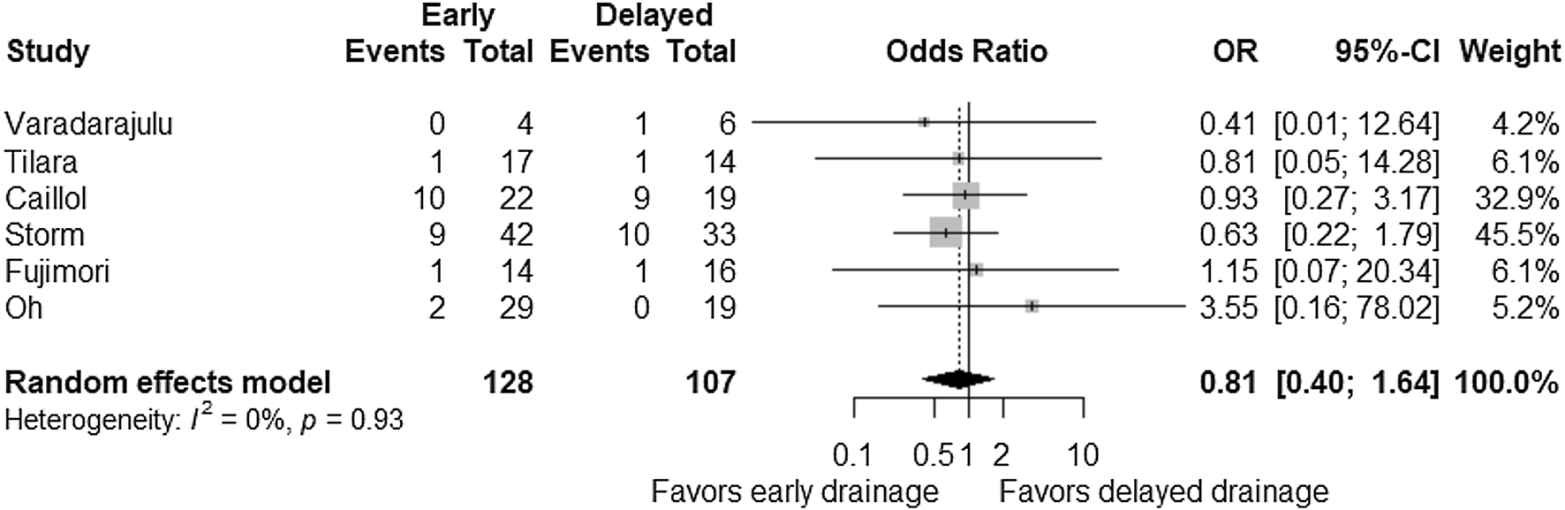
Comparison of adverse events between early and delayed EUS-guided interventions. Odds ratio (OR) for early intervention compared with delayed intervention is presented for each study (center of gray square) with 95% confidence interval (CI; horizontal line). Summary OR based on a meta-analysis via the random-effect model is presented at the bottom (center of black diamond) with 95% CI (the width of black diamond). *P* value for the *Q* statistic for between-study heterogeneity is shown

**Fig. 3:**
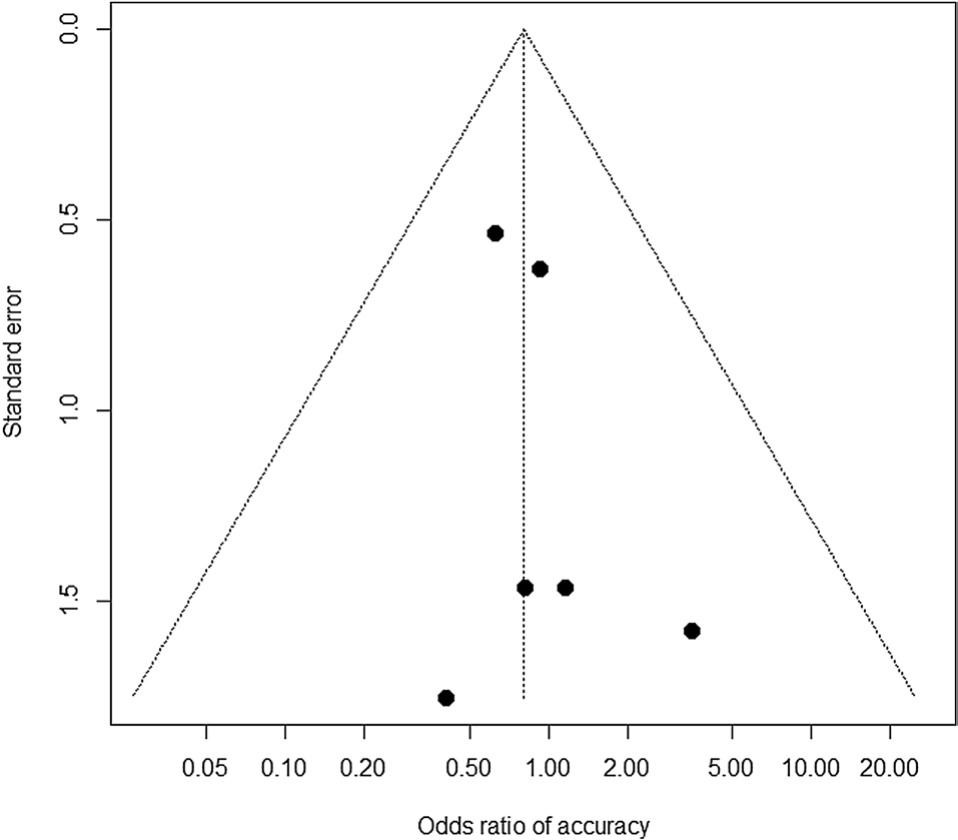
Funnel plot to examine potential publication bias in odds ratio of adverse events. Each dot indicates a respective study. Diagonal dotted lines indicate 95% confidence limits. *P* = 0.57 for Begg’s rank correlation test and *P* = 0.42 for the Egger’s linear regression test

Reported AE rates of EUS-guided drainage of POPFCs ranged from 4 to 46% (Table 2) and were comparable between early and delayed drainage groups with a pooled OR of 0.81 (95% CI 0.40–1.64; *P* = 0.55; Fig. 2). There was no evidence on heterogeneity between the studies (*P*_heterogeneity_ = 0.93 and *I*^*2*^ = 0%). Based on quantitative measurement using Egger’s test as well as visual inspection of the funnel plot, there was no significant evidence of publication bias in reporting AEs (Fig. 3). Subgroup analyses according to the timing of drainage (about two weeks and four weeks) did not show significant differences in AEs, either pooled ORs were 1.94 (95% CI 0.24–15.89; *P* = 0.54) for two weeks and 0.72 (95% CI 0.34–1.53; *P* = 0.40) for four weeks. Early drainage was not associated with the risk of bleeding with a pooled OR of 1.41 (95% CI 0.52–3.83; *P* = 0.49). Procedure-related sepsis was reported in one study: 14% and 21% in early and delayed drainage [25]. In addition, pneumonia was seen in one patient receiving delayed drainage [26]. No procedure-related mortality was reported.

### Technical and clinical success and other outcomes

**Fig. 4:**
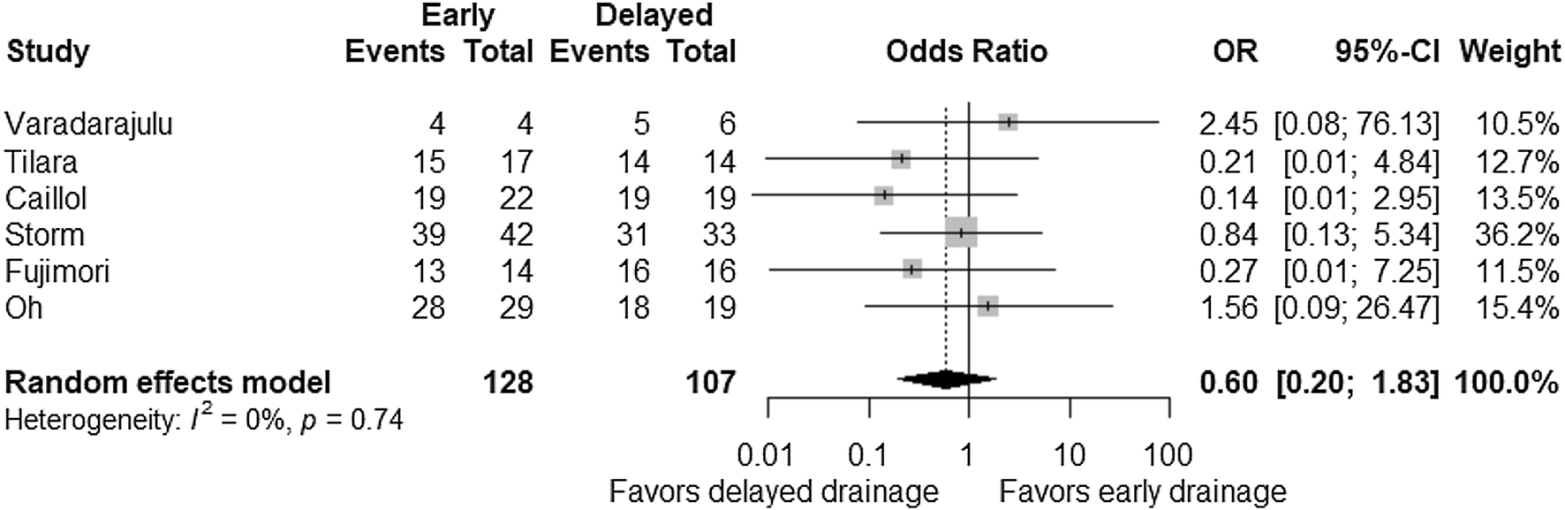
Comparison of clinical success between early and delayed EUS-guided interventions. Odds ratio (OR) for early intervention compared with delayed intervention is presented for each study (center of gray square) with 95% confidence interval (CI; horizontal line). Summary OR based on a meta-analysis via the random-effect model is presented at the bottom (center of black diamond) with 95% CI (the width of black diamond). *P* value for the *Q* statistic for between-study heterogeneity is shown

Technical success rates of early and delayed drainage groups were 100% in all six studies. Clinical success rates were reported as 90–97% and were comparable between early and delayed drainage groups with a pooled OR of 0.60 (95% CI 0.20–1.83; *P* = 0.37; Fig. 4). The rate of recurrence was evaluated in five studies but was only observed in one study [26], which reported recurrence rates of 5% and 6% in early and delayed drainage groups, respectively. The number of interventions and the length of hospital stay did not seem to be affected by the timing of EUS-guided drainage (Table 2), although these outcomes were limitedly reported [26, 27].

## Discussion

In this meta-analysis, we investigated the association of timing of EUS-guided drainage for POPFCs following surgical pancreatic resection with clinical outcomes in terms of safety and effectiveness and did not find any significant differences between early and delayed drainage. Although delayed interventions are generally recommended in PFCs after acute pancreatitis [7, 8], our meta-analysis would suggest that early EUS-guided interventions are feasible for POPFCs.

Interventions for POPFCs have shifted from invasive surgery to less invasive percutaneous or EUS-guided procedures [6]. EUS-guided drainage for POPFCs have potential advantages over percutaneous drainage, such as better quality of life, no risk of external pancreatic fistula, and less fluid loss. Meanwhile, given the nature of transmural drainage, EUS-guided interventions may contaminate POPFCs with gut microbiota and lead to bacterial peritonitis unless POPFCs are well encapsulated. In PFCs due to acute pancreatitis, early interventions can be performed safely in cases with encapsulation [29]. POPFCs were also likely to be encapsulated (> 90%) in delayed drainage group [27, 28], similar to PFCs after acute pancreatitis. In this meta-analysis, however, early EUS-guided drainage was not associated with the increased incidence of overall AEs. Infectious AEs were uncommon and did not seem to increase in early interventions, either. Although AE rates did not differ significantly by the timing of drainage, further investigation is necessary to evaluate the association of encapsulation of POPFCs with safety of EUS-guided drainage.

Differences of POPFCs from PFCs due to acute pancreatitis need comments. Given the prior surgical interventions as a cause of POPFCs, there might be some adhesions around POPFCs even in the early postoperative phase, which might reduce the risk of non-localized bacterial peritonitis. The pre-existence of necrosis in walled-off necrosis often necessitates aggressive treatment, such as irrigation and direct endoscopic necrosectomy, which may increase the risk of AEs in early drainage of PFCs due to acute pancreatitis. In addition, as some POPFCs resolve with conservative management alone, we need to identify POPFCs which need interventions to justify early drainage.

Despite recent investigations of early interventions for PFCs due to acute pancreatitis, there are some concerns about increased AEs [7, 8]. Patients with POPFCs, by definition, are fit for surgery and in relatively good conditions even if complicated by POPFCs. On the contrary, patients with PFCs due to pancreatitis are often in poor physical conditions because of the preceding and/or ongoing severe inflammation. Thus, the feasibility and safety of early interventions may differ between two conditions. However, EUS-guided drainage of POPFCs are often performed later than percutaneous drainage in clinical practice [30], as delayed interventions are recommended for PFCs due to acute pancreatitis [10]. Thus, our study results would give impacts on clinical management of POPFCs by showing feasibility of early EUS-guided drainage.

Limitations of our meta-analysis should be discussed. First of all, all six studies included in the analysis were retrospective ones, and the number of cases was limited. Although the indications of EUS-guided drainage were mostly pain and signs of infections, indications and timing of interventions were based on the criteria at each institution. Furthermore, data on the severity of POPFCs [3] were lacking in most studies. In recent studies on EUS-guided drainage for PFCs after acute pancreatitis, encapsulations were discussed in relation to the timing of interventions [29]. However, this concept of encapsulations has not been established in POPFCs, and limited data were available in the studies included in the analysis as discussed above. We conducted exploratory subgroup analyses of acute (< 2 weeks) EUS-guided drainage and did not find significant differences in AEs. However, only 68 cases in 2 studies were included in this subgroup analysis, and further investigation in a large cohort is mandatory to confirm how early we can intervene POPFC by EUS.

In conclusion, our meta-analysis suggests POPFCs can be managed by early EUS-guided drainage without an increase in AEs but the evidence is uncertain. Given potential benefits from early drainage (e.g., early recovery of patients), prospective studies are desired to validate our finding of the safety and effectiveness of early EUS-guided interventions for POPFCs.

### Supplementary Information

Below is the link to the electronic supplementary material.

Supplementary file1 (DOCX 66 kb)

Supplementary file2 (DOCX 32 kb)
