# Supplementary material for: Early versus delayed EUS-guided drainage for postoperative pancreatic fluid collections: a systematic review and meta-analysis
Source: Surg Endosc. 2023 Nov 28;38(1):47–55. doi: 10.1007/s00464-023-10568-y (PMC10776699; doi:10.1007/s00464-023-10568-y)
Supplement: Supplementary file 1 — Supplementary file1 (DOCX 66 kb) [file 464_2023_10568_MOESM1_ESM.docx]

Supplementary Table 1. Strategies of database search for studies reporting clinical outcomes of endoscopic ultrasound-guided treatment of postoperative pancreatic fistula

| Database | Search terms and limitations |
| --- | --- |
| PubMed  (*n* = 237) | Searched on 12/20/2022  ("endoscopy"[Title/Abstract] OR "endoscopic"[Title/Abstract] OR "eus"[Title/Abstract]) AND ("drainage"[Title/Abstract] OR "stent"[Title/Abstract] OR "treatment"[Title/Abstract] OR “management”[Title/Abstract]) AND ("postoperative"[Title/Abstract] OR "postsurgical"[Title/Abstract] OR “pancreatectomy” [Title/Abstract] OR “pancreatic surgery”[Title/Abstract]) AND ("pancreatic fistula"[Title/Abstract] OR “fluid collection”[Title/Abstract) AND (“1990/1/1” [Date - Publication] : “2022/12/19” [Date - Publication])AND (English[Language]) |
|  |  |
| Embase  (*n* = 215) | Searched on 1/4/2023  (endoscopy:ab,ti or endoscopic:ab,ti or eus:ab,ti or endosonography:ab,ti) and (drainage:ab,ti or stent:ab,ti or treatment:ab,ti or management:ab,ti) and (postoperative:ab,ti or postsurgical:ab,ti or pancreatectomy:ab,ti or ‘pancreatic surgery’:ab,ti) and (‘pancreatic fistula’:ab,ti or ‘fluid collection’:ab,ti)  Limitations: “English”, not “conference abstract”, “human”, and “published between 1990 and 2022” |
|  |  |
| Web of Science  (*n* = 657) | Searched on 12/20/2022  (TI=((endoscopy) OR (endoscopic) OR (EUS)) OR AB=((endoscopy) OR (endoscopic) OR (EUS))) AND (TI=((drainage) OR (stent) OR (treatment) OR (management)) OR AB=((drainage) OR (stent) OR (treatment) OR (management))) AND (TI=((postoperative) OR (postsurgical) OR (pancreatectomy) OR (pancreatic surgery)) OR AB=((postoperative) OR (postsurgical) OR (pancreatectomy) OR (pancreatic surgery))) AND (TI=((pancreatic fistula) OR (fluid collection)) OR AB=((pancreatic fistula) OR (fluid collection)))  Limitations: “published date between 1999/1/1 and 2022/12/19” |
|  |  |
| Cochrane Library (*n* = 306) | Searched on 12/20/2022  (endoscopy or endoscopic or EUS) and (drainage or stent or treatment or management) and (postoperative or postsurgical or pancreatectomy or pancreatic surgery) and (pancreatic fistula or fluid collection)  Word variations were searched.  Limitations: “title, abstract”, “English Language”, and “published between 1990 and 2022” |
|  |  |

Supplementary Table 2. The Newcastle-Ottawa Scale for assessment of data reporting quality of each study included in a meta-analysis

|  | Selection | Comparability | Exposure |
| --- | --- | --- | --- |
| Varadarajulu, 2009 [[23](#_ENREF_23)] | ** | * | * |
| Tilara, 2014 [[24](#_ENREF_24)] | ** | * | ** |
| Caillol, 2019 [[25](#_ENREF_25)] | ** | * | ** |
| Storm, 2020 [[26](#_ENREF_26)] | *** | * | ** |
| Fujimori, 2021 [[27](#_ENREF_27)] | *** | * | ** |
| Oh, 2022 [[28](#_ENREF_28)] | ** | * | ** |

Study can be awarded a maximum of 4 stars for Selection, 2 stars for Comparability, and 3 stars for Exposure. According to the total score (the number of stars), the quality of data reporting was categorized as poor (0-2 points), fair (3-6 points), and good (≥7 points).
